# Radiation-induced xerostomia and oncologic outcomes in nasopharyngeal carcinoma treated with comprehensive salivary gland-sparing helical tomotherapy: a prospective study of 266 patients

**DOI:** 10.3389/fonc.2026.1798736

**Published:** 2026-04-27

**Authors:** Feng Teng, Qiteng Liu, Lingling Meng, Zhongjian Ju, Xiangkun Dai, Xinxin Zhang, Lin Ma

**Affiliations:** 1 Department of Radiotherapy, Beijing Tongren Hospital, Capital Medical University, Beijing, China; 2 Department of Radiotherapy, Beijing Luhe Hospital, Affiliated to Capital Medical University, Beijing, China; 3 Department of Radiation Oncology, First Medical Center of Chinese PLA General Hospital, Beijing, China; 4 Department of Otorhinolaryngology Head and Neck Surgery, First Medical Center of Chinese PLA General Hospital, Beijing, China

**Keywords:** helical tomotherapy, nasopharyngeal carcinoma, radiation-induced xerostomia, salivary gland sparing, survival

## Abstract

**Objective:**

To evaluate whether comprehensive salivary gland–sparing helical tomotherapy (HT) can reduce radiation-induced xerostomia without compromising locoregional control or survival in patients with nasopharyngeal carcinoma (NPC).

**Methods:**

A total of 266 patients with NPC treated with HT were prospectively analyzed. A comprehensive salivary gland–sparing planning strategy, including preservation of the parotid glands and other salivary structures whenever clinically feasible, was applied to minimize irradiation of salivary glands. Xerostomia was assessed using patient-reported outcome measures during follow-up. Acute and late toxicities were graded according to the RTOG/EORTC criteria. Overall survival (OS), cancer-specific survival (CSS), locoregional recurrence-free survival (LRRFS), and distant metastasis-free survival (DMFS) were estimated using the Kaplan–Meier method.

**Results:**

With a median follow-up of 70.5 months, patients showed significant improvement in xerostomia symptoms. Late xerostomia was observed in 26 patients (9.8%) with Grade I–II, and only one patient (0.4%) developed Grade III xerostomia. The 1-, 3-, and 5-year overall survival (OS) rates were 95.9%, 86.8%, and 81.6%, respectively, while the corresponding cancer-specific survival (CSS) rates were 98.1%, 92.6%, and 90.2%. Locoregional control remained excellent, with a locoregional recurrence rate of 7.5% and a 5-year locoregional recurrence-free survival (LRRFS) of 92.1%. No grade 4 acute or late toxicity was observed. Multivariate Cox regression analysis demonstrated that age was significantly associated with survival outcomes (OS: p = 0.01; CSS: p = 0.01).

**Conclusions:**

Comprehensive salivary gland-sparing helical tomotherapy reduces radiation-induced xerostomia without compromising locoregional control or survival, supporting its oncologic safety in NPC radiotherapy.

**Clinical trial registration:**

https://www.chictr.org.cn/showproj.html?proj=17360, identifier ChiCTR-ONN-17010597.

## 1 Introduction

Xerostomia is a common and debilitating late toxicities of radiotherapy for Nasopharyngeal Carcinoma (NPC). Despite advances in intensity-modulated radiotherapy (IMRT), clinically significant xerostomia remains frequent because the parotid and other salivary glands are highly radiosensitive (1–3). Moderate xerostomia occurs in approximately 40–60% of patients and severe xerostomia in 15–30% after conventional radiotherapy; even with IMRT, 20–40% of patients continue to experience Grade ≥2 xerostomia during long-term follow-up (4, 5).

Preserving salivary gland function has therefore become a key objective in modern radiotherapy planning. The joint ISOO/MASCC/ASCO clinical practice guidelines (6) strongly recommend the use of tissue-sparing radiation modalities as a primary preventive strategy. Comprehensive salivary gland sparing involves reducing radiation exposure to multiple salivary structures, including the parotid, submandibular, and sublingual glands, as well as minor salivary glands within the oral cavity, whenever clinically feasible. Helical tomotherapy (HT), with its highly conformal rotational delivery, offers enhanced ability to spare the salivary gland while maintaining target coverage (7).

Our previous clinical observations further showed that patients receiving lower radiation doses to the major salivary glands experienced more substantial xerostomia improvement over time, with more than 60% recovering to near-baseline levels at 12 months and nearly 70% by 18 months (7). These findings highlight the clinical value of salivary preservation.

However, concerns remain regarding whether salivary gland-sparing strategies might compromise locoregional tumor control, particularly in cases with extensive nodal involvement. High-quality prospective evidence confirming long-term oncologic safety is still limited (8–10).

Therefore, the present study evaluates whether comprehensive salivary gland–sparing helical tomotherapy can reduce xerostomia without compromising locoregional control or survival in patients with NPC. This study provides real-world evidence supporting the safe incorporation of salivary-sparing strategies into routine radiotherapy.

## 2 Materials and methods

### 2.1 Eligibility criteria

Patients with histologically or cytologically confirmed, newly diagnosed nasopharyngeal carcinoma (NPC) who were scheduled to receive definitive radiotherapy were eligible for inclusion. All patients had a Karnofsky Performance Status (KPS) score ≥70, and had no prior radiotherapy to the head and neck region. Pretreatment contrast-enhanced computed tomography (CT) and/or magnetic resonance imaging (MRI) was required for accurate delineation of targets and salivary glands. Patients needed to be medically fit to undergo helical tomotherapy (HT) and able to comply with scheduled follow-up assessments.

Exclusion criteria included pre-existing severe xerostomia or systemic diseases affecting salivary gland function (e.g., Sjögren syndrome), uncontrolled comorbidities that would preclude safe radiotherapy delivery, incomplete imaging data preventing salivary gland evaluation, treatment discontinuation due to intolerance or disease progression, or inability to complete follow-up. Patients who had previously received chemotherapy or radiotherapy to the head and neck were excluded.

Written informed consent was obtained from all participants prior to enrollment. The study was approved by the Ethics Committee of the Chinese PLA General Hospital.

### 2.2 Intensity modulated radiation technique

All patients underwent CT-based simulation in the supine position with a head-neck–shoulder thermoplastic mask for immobilization. A contrast-enhanced CT scan was acquired from the skull vertex to the carina with 3-mm slice thickness. CT datasets were transferred to the TomoTherapy Hi-Art planning system (Accuray, USA) for contouring and treatment planning. When available, diagnostic MRI was fused with planning CT for accurate delineation of the primary tumor, nodal disease, and salivary glands. Target volumes were defined according to the ICRU Report 83 guidelines. The gross tumor volume (GTV) included the primary tumor and metastatic lymph nodes identified on CT/MRI. The clinical target volume (CTV) encompassed the GTV and areas at risk of microscopic spread, including high-risk mucosal sites and nodal regions, depending on tumor location. A 3–5 mm margin was added to the CTV to generate the planning target volume (PTV), adjusted as needed to account for anatomical proximity to critical organs at risk (OARs).

OARs included the bilateral parotid glands, submandibular glands, oral cavity, spinal cord, brainstem, mandible, optic structures, temporomandibular joints, and larynx. Particular emphasis was placed on sparing the parotid glands while ensuring adequate PTV coverage. Treatment plans were optimized to deliver at least 95% of the prescribed dose to 95% of the PTV while minimizing dose to OARs.

All patients were treated with helical tomotherapy using daily megavoltage computed tomography (MVCT) for image-guided setup verification. The prescribed radiation dose ranged from 66–70 Gy to the primary tumor and involved lymph nodes and 54–60 Gy to elective nodal regions, delivered in 30–35 fractions (five fractions per week).

### 2.3 Organ-at-risk contouring and dose constraints

OARs included the bilateral parotid glands, submandibular glands, oral cavity, spinal cord, brainstem, mandible, optic structures, temporomandibular joints, and larynx. Special emphasis was placed on sparing the parotid glands, as they are critical in reducing xerostomia. The submandibular glands and oral cavity were also prioritized for sparing whenever clinically feasible. Dose constraints for these OARs were set based on previously published studies and ROC curve analysis. For example, the parotid glands were constrained to a mean dose (Dmean) of <29.12 Gy, the submandibular glands to <29.29 Gy, and the oral cavity to <31.44 Gy, to minimize radiation-induced xerostomia while ensuring adequate tumor coverage.

### 2.4 Toxicity and measurement

Acute and late toxicities were evaluated weekly during treatment and at each follow-up visit according to the RTOG/EORTC criteria. Xerostomia was assessed using patient-reported xerostomia questionnaire (XQ) scores (**Table 1**) at baseline and at 1, 3, 6, 12, and 18 months after radiotherapy. Moderate-to-severe xerostomia was defined as Grade ≥2. Patients were closely monitored for mucositis, dermatitis, dysphagia, hematologic toxicity, and treatment-related adverse events. Dose constraints and treatment continuation were determined by the attending radiation oncologist according to institutional protocol.

**Table 1:** Xerostomia questionnaire (XQ).

1. What is the overall comfort of your mouth?
2. Does your mouth feel dry when eating?
3. Do you have difficulty swallowing because of dry mouth?
4. Do you have difficulty chewing because of dry mouth?
5. Do you have problems with speech because of dry mouth?
6. Do you have problems with sleeping because of dry mouth?
7. Do you need drink water when swallowing dry food?
8. How often do you need to drink water during one hour to keep your mouth comfortable? Less than once, once, two–three times, or more than three times?
9. Do you feel the amount of saliva in your mouth is no, too little, too much, or adequate?
10. Has you taste changed because of salivary gland function?
Patients rated each item on a scale from 0 to 3. The higher the score, the worse the xerostomia.

### 2.5 Endpoints and statistics

The primary endpoint was the incidence and recovery of xerostomia after comprehensive salivary gland–sparing radiotherapy. Secondary endpoints included acute and late toxicities, locoregional recurrence, locoregional recurrence-free survival (LRRFS), distant metastasis-free survival (DMFS), overall survival (OS)., as well as the incidence of xerostomia and other acute/late toxicities.

The survival endpoints were defined as follows:

OS: the time from the date of enrollment to the date of death from any cause or the last follow-up.
CSS: the time from the date of enrollment to the date of death caused specifically by nasopharyngeal carcinoma.
LRRFS: the interval between enrollment and the first documented locoregional recurrence.
DMFS: the time from enrollment to the first occurrence of distant metastasis.

Survival outcomes were estimated using the Kaplan–Meier method, and differences were compared using the log-rank test. Continuous variables were summarized as mean ± standard deviation or median (interquartile range), and categorical variables as frequencies and percentages. Between-group differences were assessed using the independent t-test, Mann–Whitney U test, χ² test, or Fisher’s exact test, as appropriate. A p-value <0.05 was considered statistically significant. Statistical analyses were performed using SPSS version 25.0 (IBM Corp., Armonk, NY, USA). Multivariate Cox regression was used to identify independent prognostic factors, adjusting for potential confounders such as age, gender, and disease stage.

## 3 Results

### 3.1 Clinical characteristics

**Figure 1:**
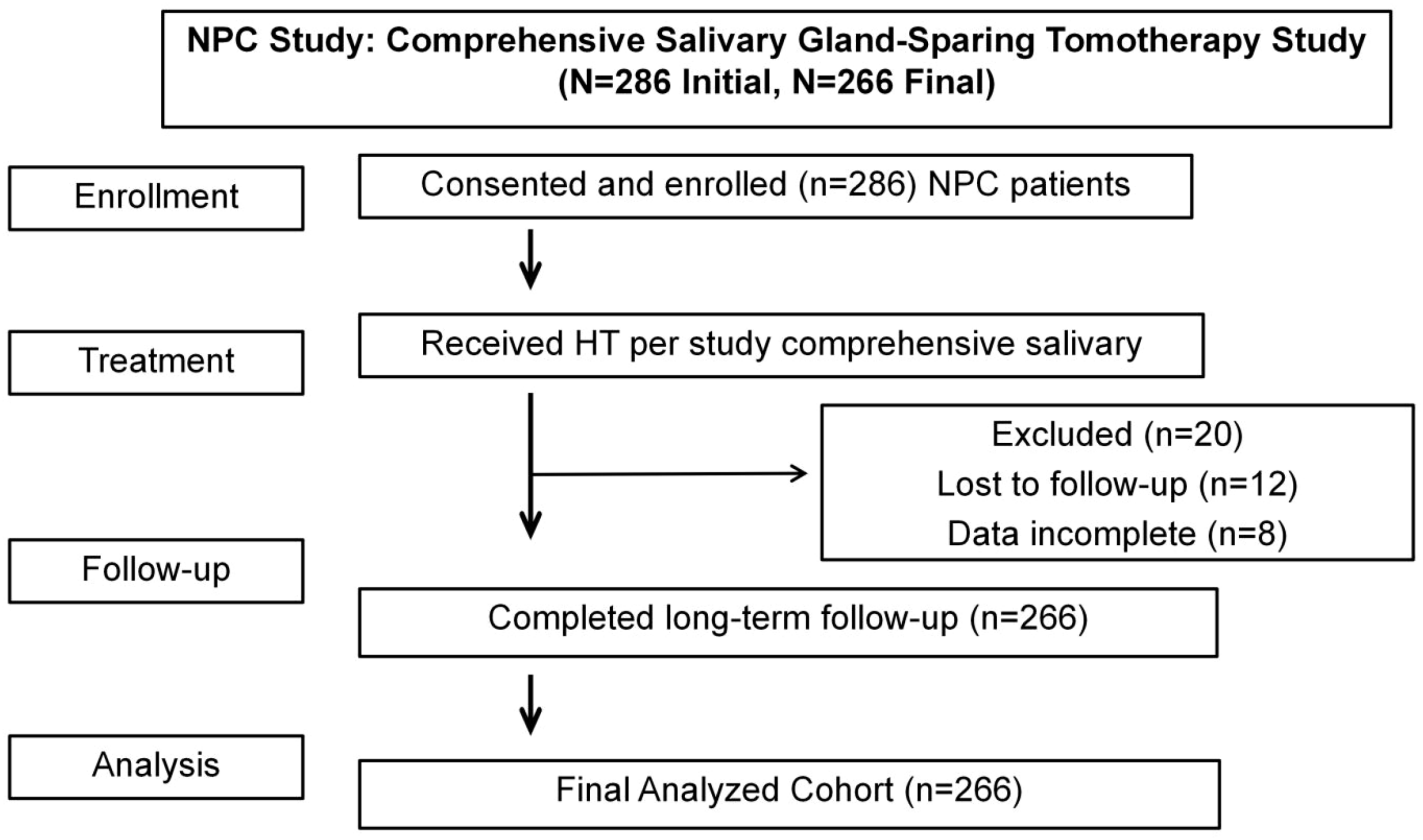
CONSORT flow diagram of patient enrollment and follow-up in this study.

This study included 266 patients with nasopharyngeal carcinoma (NPC) treated with salivary gland-sparing helical tomotherapy. From February 2016 to February 2019, 286 patients were enrolled in the study, with 20 patients lost to follow-up during the study period. Therefore, a total of 266 patients were included in the final analysis (as shown in **Figure 1**). The majority of patients were male, with 192 males (72.2%) compared to 74 females (27.8%). The median age of the patients was 51 years (interquartile range [IQR]: 41–59 years). Regarding tumor staging, based on the UICC/AJCC (7th edition, 2010) criteria, most patients were diagnosed with advanced-stage disease. Stage III was the most common, with 141 patients (53.0%); followed by Stage IVb (24.1%) and Stage IVa (17.7%). Only 5.3% of patients were diagnosed with Stage II. In terms of T-stage, T2 was the most common, accounting for 48.5%, followed by T3 at 22.6%. In N-stage, N2 was the most common, comprising 44.0%, followed by N3 at 24.4%. Detailed baseline patient and tumor characteristics are summarized in **Table 2**.

**Table 2:** Clinicopathological characteristics of patients with nasopharyngeal carcinoma (NPC).

Characteristic	Overall n=266
Sex, men, n (%)	192 (72.2)
Sex, women, n (%)	74 (27.8)
Age, years, (median (IQR))	51 (41–59)
T-stage, n (%)	
T1	21 (7.9)
T2	129(48.5)
T3	60(22.6)
T4a	35(13.2)
T4b	21(7.9)
N-stage, n (%)	
N0	27(10.2)
N1	57(21.4)
N2	117(44.0)
N3	65(24.4)
UICC/AJCC (2010), n (%)	
Stage II	14 (5.3)
Stage III	141 (53.0)
Stage IVa	47(17.7)
Stage IVb	64 (24.1)
Treatment modality, n (%)	
Chemoradiotherapy (CCRT)	255(95.9)
RT alone	11 (4.1)

### 3.2 Treatment outcomes

During a median follow-up of 70.5 months (95% CI, 69.0–73.3 months; range, 3.9-95.6 months; IQR, 61.8–80.0 months), treatment outcomes were favorable across the entire cohort. A total of 72 patients (27.1%) died during follow-up, including 32 tumor-related deaths. Disease progression occurred in 50 patients, among whom 20 (7.5%) developed locoregional recurrence and 30 (11.3%) experienced distant metastasis. The lung was the most common metastatic site, followed by bone and liver. All locoregional recurrences occurred within the primary tumor bed and/or initially involved nodal regions. Notably, no marginal failures were observed adjacent to the spared salivary glands, including the parotid glands, submandibular glands, or oral cavity.

**Figure 2:**
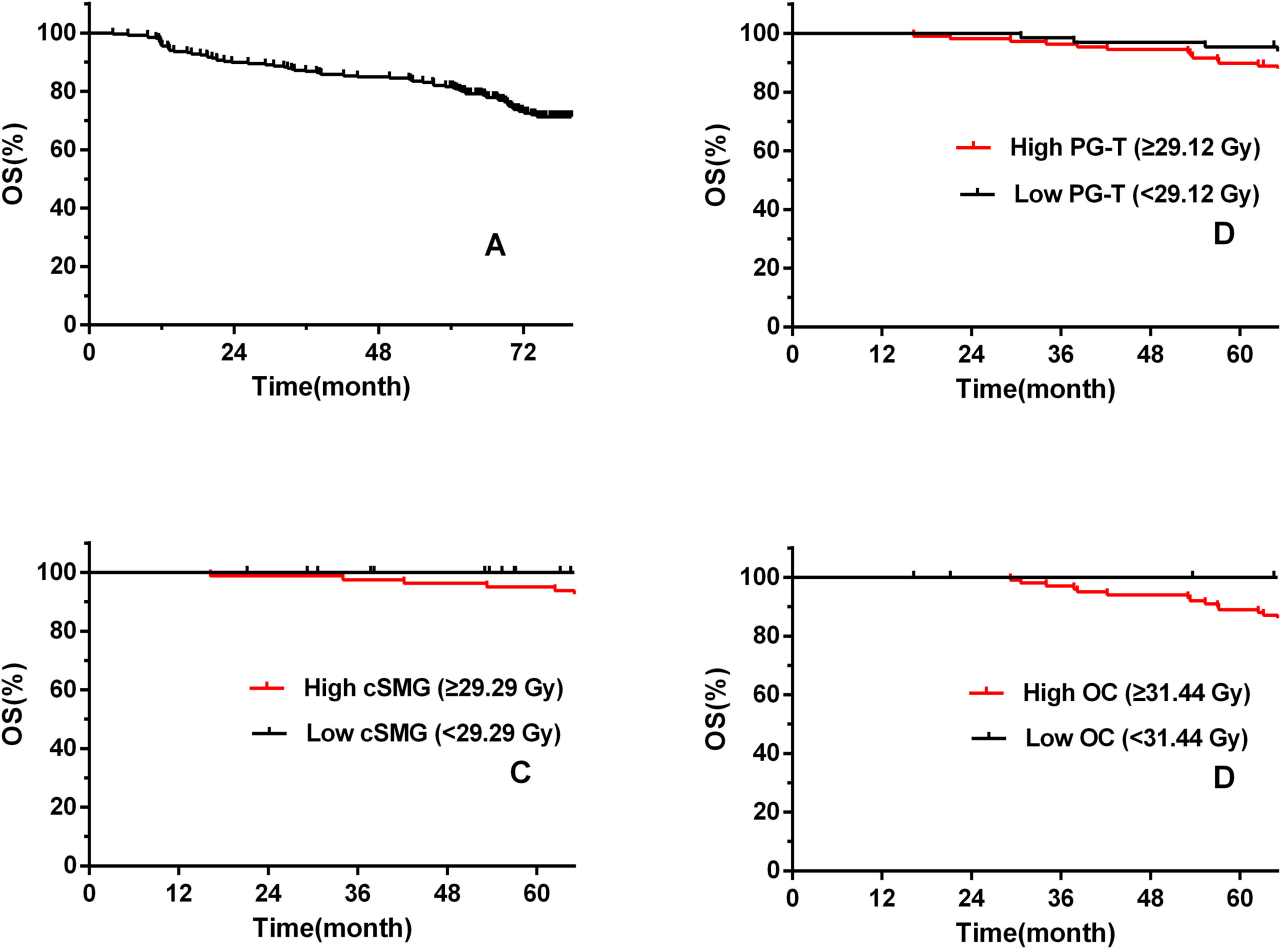
Kaplan–Meier curves of overall survival (OS) in patients with nasopharyngeal carcinoma treated with comprehensive salivary gland–sparing helical tomotherapy. **(A)** Overall survival (OS) of the entire cohort. **(B)** OS stratified by mean total parotid gland dose (PG-T < 29.12 Gy vs. ≥ 29.12 Gy). **(C)** OS stratified by mean combined submandibular gland dose (cSMG < 29.29 Gy vs. ≥ 29.29 Gy). **(D)** OS stratified by mean oral cavity dose (OC < 31.44 Gy vs. ≥ 31.44 Gy).

Long-term tumor control remained excellent. The 1-, 3-, and 5-year overall survival (OS) rates were 95.9%, 86.8%, and 81.6%, respectively. The 1-, 3-, and 5-year cancer-specific survival (CSS) rates were 98.1%, 92.6%, and 90.2%, while the corresponding locoregional recurrence-free survival (LRRFS) rates were 98.9%, 94.3%, and 92.1%. The 1-, 3-, and 5-year distant metastasis-free survival (DMFS) were 94.7%, 91.9%, and 88.6%, respectively (**Figure 2**).

3. Oncologic safety of comprehensive salivary gland–sparing HT

Patients receiving lower mean parotid and submandibular gland doses (PG-T <29.12 Gy, cSMG <29.29 Gy, OC <31.44 Gy) showed significantly faster improvement in xerostomia beginning at 6 months, with the difference persisting and widening through 12 and 18 months (7). By 18 months, 69.6% of patients achieved near-baseline salivary function, and only one patient(0.4%) experienced Grade III xerostomia. Importantly, comprehensive salivary gland-sparing helical tomotherapy did not compromise tumor control or survival. All three- and five-year survival metrics were consistent with published historical standards for non-comprehensive salivary gland–sparing radiotherapy (**Figure 2**).

Subgroup analyses revealed no significant differences in OS, CSS, or LRRFS with respect to sex, stage, or salivary gland dose (all p > 0.05). However, multivariate Cox regression analysis showed that age was significantly associated with survival outcomes (OS: p = 0.01; CSS: p = 0.01). Detailed results are provided in **Tables 3**, **4**.

**Table 3:** Hazard ratios from univariate cox regression models for survival outcomes in patients.

		OS			CSS			LRRFS			DMFS	
Variable	HR	95% CI	P	HR	95% CI	P	HR	95% CI	P	HR	95% CI	P
OC Dmean	1.02	0.916-1.142	0.68	0.90	0.722-1.125	0.36	0.84	0.634-1.112	0.22	1.01	0.848-1.191	0.96
PG-T Dmean	1.07	0.97-1.172	0.18	1.10	0.965-1.252	0.16	1.11	0.937-1.305	0.23	1.01	0.851-1.188	0.95
cSMG Dmean	0.98	0.952-1.013	0.25	1.00	0.962-1.043	0.94	0.99	0.928-1.048	0.64	0.98	0.937-1.027	0.42
Age	1.08	1.045-1.123	0.01	1.06	1.008-1.119	0.03	1.02	0.964-1.078	0.50	1.04	0.994-1.082	0.10
Gender	1.05	0.492-2.247	0.90	0.71	0.191-2.613	0.60	2.80	0.625-12.512	0.18	0.16	0.021-1.231	0.08
Stage (II+III vs IV)	1.60	0.766-3.34	0.21	0.95	0.286-3.162	0.94	0.31	0.037-2.579	0.28	0.98	0.328-2.917	0.97

**Table 4:** Hazard ratios from multivariate cox regression models for survival outcomes in patients.

		OS			CSS			LRRFS			DMFS	
Variable	HR	95% CI	P	HR	95% CI	P	HR	95% CI	P	HR	95% CI	P
OC Dmean	0.99	0.887-1.128	0.99	0.83	0.629-1.086	0.17	0.80	0.568-1.123	0.20	0.98	0.813-1.189	0.86
PG-T Dmean	1.07	0.971-1.197	0.15	1.16	1.001-1.335	0.05	1.13	0.96-1.327	0.14	1.04	0.865-1.239	0.70
cSMG Dmean	0.99	0.966-1.034	0.97	1.02	0.981-1.069	0.27	1.00	0.943-1.067	0.92	0.99	0.941-1.036	0.61
Age	1.08	1.046-1.128	0.01	1.08	1.017-1.141	0.01	1.04	0.97-1.119	0.27	1.03	0.985-1.074	0.20
Gender	1.41	0.644-3.105	0.38	0.69	0.175-2.725	0.60	2.40	0.474-12.123	0.29	0.17	0.022-1.302	0.09
Stage (II+III vs IV)	1.64	0.767-3.506	0.20	0.86	0.252-2.961	0.82	0.35	0.041-2.937	0.33	0.94	0.303-2.938	0.92

### 3.3 Late toxicities and complications

During long-term follow-up, late treatment-related toxicities were generally mild and manageable. Among the 266 patients, 142 (53.4%) reported no late adverse effects at the final assessment. The most frequently observed late toxicity was radiation-related otologic injury, including hearing loss, otitis media, or tinnitus, which occurred in 61 patients (22.9%). Most of these otologic symptoms were mild and did not require invasive intervention.

Late xerostomia occurred in 26 patients (9.8%) with Grade I–II, and only one patient (0.4%) experienced Grade III xerostomia; no Grade 4 xerostomia was observed. Swallowing difficulty was rare, with only two patients (0.8%) reporting late dysphagia, none of whom required enteral nutritional support. In addition, 34 patients (12.8%) reported other late adverse events, such as headache, visual disturbance, or thyroid dysfunction, all of which were mild and controllable with conservative management. Detailed results are provided in **Tables 5**, **6**.

**Table 5:** Late radiation related toxicities of 266 patients.

Late complications	No.of patients	
	Grade I/II	Grade III
Xerostomia	26	1
Hearing impairment	58	3
Subcutaneous fibrosis	34	8
Hypothyroidism	13	0
Trismus	2	0
Temporal lobe necrosis	11	0
Cranial nerve palsies	6	0
Eyeball damage	4	0

**Table 6:** Summary of studies on survival data and xerostomia incidence in head and neck cancer under different radiotherapy techniques.

Study/evidence	Study design & population	Radiotherapy technique/strategy	Survival/tumor control outcomes	Xerostomia outcomes
Current Study (NPC, n=266)	Prospective cohort; Median follow-up 70.5 months	Comprehensive salivary gland-sparing Helical Tomotherapy (HT); parotid, submandibular, and oral cavity structures preserved when clinically feasible	OS: 1-y 95.9%, 3-y 86.8%, 5-y 81.6%; LRRFS: 5-y 92.1%	Late Xerostomia: Grade 1-2 (9.8%, 26/266); Grade 3 (0.4%, 1/266); No Grade 4
PARSPORT (HNC, n=94)	Multi-center Phase III RCT	Parotid-sparing IMRT vs 3D-CRT	2-y OS: IMRT 78% vs 3D-CRT 76% (P = 0.68); 2-y LRPFS: IMRT 78% vs 3D-CRT 80% (P = 0.34)	2-y≥Grade 2 xerostomia: 29% vs 83% (P<0.0001)
RTOG 0225 (NPC, n=68)	Multi-center Phase II; Stage I-IVB	IMRT	2-y OS: 80.2%; 2-y locoregional control: 89.3%	1-y Grade 2 xerostomia: 13.5%; late Grade 3 xerostomia: 3.1%; No Grade 4
Kam et al.(NPC, n=60)	Prospective randomized trial; T1-2bN0-1M0	Parotid-sparing IMRT vs 2D-RT	Not reported	1-y observer-rated severe xerostomia: IMRT 39.3% vs 2D-RT 82.1% (P = 0.001)

## 4 Discussion

Radiation-induced xerostomia remains one of the most common and debilitating late toxicities in patients with NPC receiving radiotherapy. Although advances in radiotherapy techniques have improved dose conformity and organ-at-risk protection, a substantial proportion of long-term survivors continue to experience clinically meaningful xerostomia, which adversely affects speech, swallowing, taste, oral health, and overall quality of life. Consequently, reducing xerostomia while maintaining oncologic safety has become a major objective in contemporary head and neck radiotherapy (6).

The landmark PARSPORT phase III randomized controlled trial provided strong evidence supporting parotid-sparing IMRT. At 24 months after treatment, the incidence of ≥Grade 2 xerostomia was 29% with IMRT vs. 83% with conventional radiotherapy (p < 0.0001), demonstrating the substantial clinical benefit of parotid preservation (3). These results established salivary gland sparing as a key objective in modern radiotherapy planning.

However, accumulating evidence indicates that sparing the parotid glands alone may be insufficient to fully prevent xerostomia. The submandibular glands, sublingual glands, and minor salivary glands within the oral cavity contribute substantially to unstimulated and baseline salivary secretion, particularly under resting conditions (4). Radiation dose to these structures has been closely associated with persistent dry mouth and delayed recovery of salivary function (11).

Comprehensive salivary gland sparing, which aims to preserve multiple major and minor salivary glands simultaneously, has emerged as an important objective in modern radiotherapy for NPC. Helical tomotherapy (HT), with its continuous rotational delivery, fine beam modulation, and superior dose conformality, offers a distinct technical advantage for implementing this strategy while maintaining adequate target coverage (7, 9). In the present prospective study, a comprehensive salivary gland–sparing approach was systematically applied whenever clinically feasible.

A prior prospective observational study by Teng et al. (7)provided early clinical evidence that comprehensive sparing of the bilateral parotids (PG-T), contralateral submandibular gland (cSMG), and oral cavity (OC) using helical tomotherapy could mitigate xerostomia without compromising early locoregional control. In that cohort (n=175) with serial assessments of saliva flow and xerostomia questionnaire scores, ROC analysis identified clinically actionable Dmean thresholds for PG-T, cSMG, and OC (29.12, 29.29, and 31.44 Gy, respectively). Notably, the xerostomia benefit became more apparent over time, with significant separation of symptom trajectories at 12 and 18 months. Our current long-term analysis extends and strengthens these observations by confirming durable tumor control and sustained toxicity benefits over a substantially longer follow-up. These findings provide direct dose–response evidence that maintaining salivary gland and oral cavity doses below approximately 30 Gy is associated with meaningful long-term xerostomia relief.

A major concern regarding aggressive organ-sparing strategies is the potential compromise of locoregional tumor control, particularly in patients with advanced-stage disease or extensive nodal involvement. In the present study, locoregional recurrences were not concentrated in regions adjacent to the spared salivary glands. No marginal or gland-adjacent failures involving the parotid glands, submandibular glands, or oral cavity were observed. Prior studies (11) have demonstrated that constraining the contralateral submandibular gland mean dose to ≤39 Gy does not increase the risk of contralateral level IB failure in appropriately selected patients. This is further reinforced by the expert consensus in the latest IG-2024 update (12), which clarifies that routine coverage of the SMGs is not mandatory for N0 or non-involved cases. Our results, showing a 70.5-month median follow-up with excellent locoregional control, provide clinical validation for this selective sparing strategy in the era of Helical Tomotherapy. These findings provide spatial evidence that comprehensive salivary gland–sparing helical tomotherapy can be safely implemented without increasing the risk of geographic miss or gland-adjacent recurrence.

The present study provides reassuring evidence that comprehensive salivary gland–sparing HT does not adversely affect oncologic outcomes. With a median follow-up exceeding 70 months, the long-term oncologic outcomes in this study were highly favorable. The 5-year OS and CSS rates of 81.6% and 90.2%, respectively, as well as a 5-year LRRFS of 92.1%, compare favorably with historical series of NPC treated with conventional IMRT or non–salivary gland sparing approaches. Importantly, the low locoregional recurrence rate (7.5%) observed in this cohort provides strong evidence that comprehensive salivary gland sparing does not compromise tumor control, even in patients with advanced T and N stages.

Subgroup and multivariate analyses further confirmed the oncologic safety of this approach. Salivary gland dose parameters were not associated with inferior overall survival, cancer-specific survival, or locoregional recurrence-free survival, whereas age remained the primary prognostic factor influencing survival outcomes. Although the mean total parotid gland dose showed a borderline association with cancer-specific survival in multivariate analysis (P = 0.05), the effect size was small and the confidence interval was close to unity; therefore, this finding should be interpreted with caution. These findings suggest that comprehensive salivary gland sparing can be safely integrated into modern radiotherapy planning without increasing the risk of disease recurrence.

Recent high-level evidence, such as the SPLS-IMRT phase II trial (13), has demonstrated that refined sparing of salivary gland subunits can reduce Grade 3 xerostomia to 0%, shifting the paradigm towards more granular and comprehensive organ preservation. In alignment with this trend, late treatment-related toxicities in this cohort were generally mild and manageable. No grade 4 acute or late adverse events were observed, and the incidence of severe late xerostomia, dysphagia, or other catastrophic complications was extremely low. This outcome is highly consistent with the findings reported by the SPLS-IMRT trial, further reinforcing that a multi-gland sparing strategy, facilitated by the superior conformality of Helical Tomotherapy, is a highly effective approach for long-term functional recovery. From a survivorship perspective, reducing xerostomia without increasing late morbidity is of substantial clinical importance for long-term quality of life in NPC survivors.

Regarding other late toxicities, hearing impairment and subcutaneous fibrosis were the most prevalent adverse events in our cohort (**Table 4**). The incidence of late hearing impairment was 21.8% (58/266). Given the inherent anatomical proximity of the nasopharyngeal PTV to the Eustachian tubes and cochleae, minimizing Grade I–II hearing loss remains a formidable clinical challenge that warrants further investigation in our future protocols. Nevertheless, severe (Grade 3) hearing loss was restricted to only 1.1% (3/266) of patients. This outcome underscores the technical superiority of Helical Tomotherapy (HT) in achieving a steep dose fall-off at the interface between the target volume and the auditory apparatus. Similarly, while late subcutaneous fibrosis was observed in 12.8% of patients, the incidence of Grade 3 symptoms was remarkably low at 3.0% (8/266). By optimizing dose distribution to limit the “low-dose bath” within the cervical soft tissues, our comprehensive sparing strategy effectively mitigated the risk of severe neck stiffness and trismus, further reinforcing the holistic safety and therapeutic index of this approach for long-term NPC survivors.

Several limitations should be acknowledged. This study is a single-center analysis, which may limit the generalizability of the findings. Future multi-center studies should be conducted to confirm the results and expand our understanding of the efficacy of comprehensive salivary gland-sparing Helical Tomotherapy across diverse patient populations. In addition, xerostomia assessment was primarily based on patient-reported outcomes rather than objective salivary flow measurements.

## Conclusion

Comprehensive salivary gland-sparing Helical Tomotherapy offers a promising approach in reducing radiation-induced xerostomia in patients with nasopharyngeal carcinoma, while maintaining excellent oncologic outcomes. This strategy should be considered an integral part of modern radiotherapy for NPC patients, aiming to improve both survival and quality of life.

## Data Availability

The original contributions presented in the study are included in the article/Supplementary Material. Further inquiries can be directed to the corresponding author.
